# Application of the CRISPR/Cas System in Pathogen Detection: A Review

**DOI:** 10.3390/molecules27206999

**Published:** 2022-10-18

**Authors:** Bowei Yuan, Congcong Yuan, Lulu Li, Miao Long, Zeliang Chen

**Affiliations:** Key Laboratory of Zoonosis of Liaoning Province, College of Animal Science and Veterinary Medicine, Shenyang Agricultural University, Shenyang 110866, China

**Keywords:** CRISPR/Cas, pathogen detection

## Abstract

Early and rapid diagnosis of pathogens is important for the prevention and control of epidemic disease. The polymerase chain reaction (PCR) technique requires expensive instrument control, a special test site, complex solution treatment steps and professional operation, which can limit its application in practice. The pathogen detection method based on the clustered regularly interspaced short palindromic repeats (CRISPRs) and CRISPR-associated protein (CRISPR/Cas) system is characterized by strong specificity, high sensitivity and convenience for detection, which is more suitable for practical applications. This article first reviews the CRISPR/Cas system, and then introduces the application of the two types of systems represented by Type II (cas9), Type V (cas12a, cas12b, cas14a) and Type VI (cas13a) in pathogen detection. Finally, challenges and prospects are proposed.

## 1. Introduction

In the natural environment, there are many microorganisms and parasites that can cause diseases as pathogens. An epidemic may threaten the life and health of humans and animals and cause significant economic loss. The occurrence of a serious outbreak often leads to irreversible losses or damage, so an early and rapid diagnosis of the disease is particularly important.

The traditional microbial detection techniques are smear microscopy, pathogen isolation, culture and biochemical identification and serological examination. These traditional pathogen detection methods are relatively simple and convenient, can rapidly make preliminary judgments and do not require complex instruments, while still playing an important role at the grassroots level. In molecular biology, polymerase chain reaction (PCR) has been the mainstream choice for molecular diagnostics. This technology uses DNA high-temperature denaturation, low-temperature renaturation and extension at the optimum temperature of polymerase to achieve the amplification of DNA fragments [1]. The PCR instrument manufactured based on this principle is a temperature-control device. Seen as the variable temperature amplification of PCR requires expensive instrument control, special test sites, complex solution processing steps and professional operations, it cannot be satisfactorily utilized in practice; therefore, rapid detection methods that rely on isothermal nucleic acid amplification technology have attracted increasing attention. Common isothermal amplification techniques include loop-mediated isothermal amplification (LAMP) [2], recombinase polymerase amplification (RPA) and the like.

LAMP and RPA read amplified signals using colorimetric indicators [3,4], turbidimetry [5], lateral-flow immunoassay [6,7], fluorescence [8,9] and electrochemical methods [10,11]. As the research on the CRISPR/Cas system has intensified, scientists have attempted to combine CRISPR/Cas effectors as biosensors with nucleic acid amplification technology (especially isothermal nucleic acid amplification technology) to develop a simpler, faster and more field-suitable pathogen detection method.

## 2. Introduction to the CRISPR/Cas System

**Figure 1:**
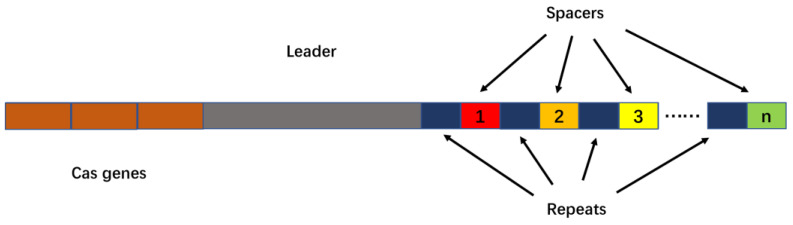
CRISPR-Cas locus structure diagram. CRISPR-Cas includes transactivating crRNA, genes encoding Cas-related proteins (Cas genes), repeat sequences and spacer sequences.

The CRISPR/Cas system is an adaptive immune defense system first discovered in Escherichia coli and used by bacteria and archaea as a defense against virus invasion. CRISPRs refers to clustered regularly interspaced short palindromic repeats, which was first described by Jansen et al. in a study of bacterial and archaeal genome sequences [12]. Its structure is shown in Figure 1.

**Figure 2:**
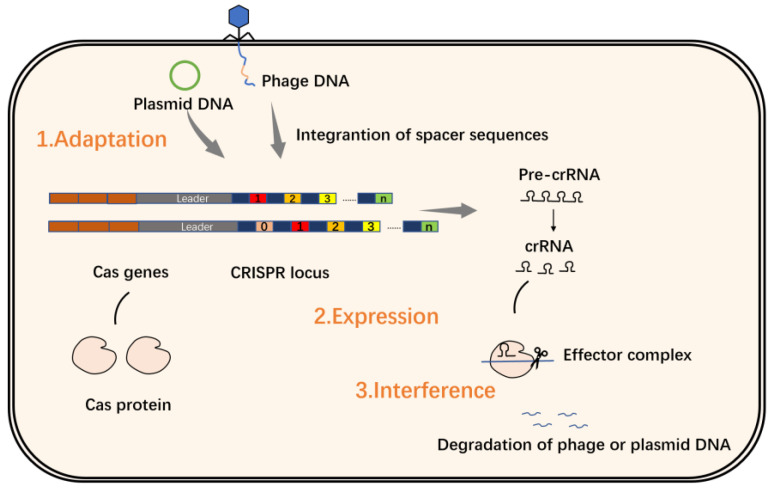
The CRISPR/Cas system in action during the adaptation phase, short DNA fragments that are homologous to the viral or plasmid sequence are integrated into the CRISPRs site as spacers; during the expression stage, the CRISPRs sequence is first transcribed into pre-crRNA, which is then processed into crRNA that matches the target sequence of the virus or plasmid; during the interference phase, the crRNA guides the Cas protein to the target sequence of the virus or plasmid that matches the spacer, forming an effector complex, and then the cleavage ability of the Cas protein degrades the phage or plasmid DNA.

CRISPR sequences consist of repeat sequences and spacer sequences. Repeat sequences are repeating palindromic sequences arranged one after the other, separated by spacer sequences, while the DNA of the spacer sequences is not identical. Research has found that spacer sequences match viral DNA, especially phage DNA [13,14,15]. The upstream gene located at the CRISPR/Cas locus is named CRISPR-associated gene, or Cas gene for short. The Cas gene is closely linked to the CRISPRs site, and the Cas protein expressed thereby plays a key role in the realization of the CRISPR/Cas system function. Cas proteins have helicase and nuclease activities that can cut DNA strands; based on these findings, scientists have proposed the theory that the CRISPR/Cas system defends against virus invasion through three stages of adaptation, expression and interference (Figure 2) [14,16,17,18].

The diversity of the CRISPR/Cas system is ascribed to its abundance of effector proteins, site structures and molecular mechanisms. According to the different types of effector proteins in the interference phase described above, the system is mainly divided into two categories, including 6 types and 33 subtypes. Class 1 systems have complex effectors consisting of multiple Cas proteins that often perform multiple functions when the system is functioning. In contrast, Class 2 systems contain only one crRNA-binding protein. Class 1 comprises Types I, III and IV, and Class 2 includes Types II, V and VI [19,20]; because Class 2 CRISPR/Cas systems contain the properties of a single multidomain effector protein, they are more widely studied and applied than Class 1 systems. Next, the application of two types of systems represented by Type II (Cas9), Type V (Cas12a, Cas12b, Cas14a) and Type VI (Cas13a) in pathogen detection is introduced.

## 3. In Vitro Pathogen Detection Based on the CRISPR/Cas System

### 3.1. Cas9

**Figure 3:**
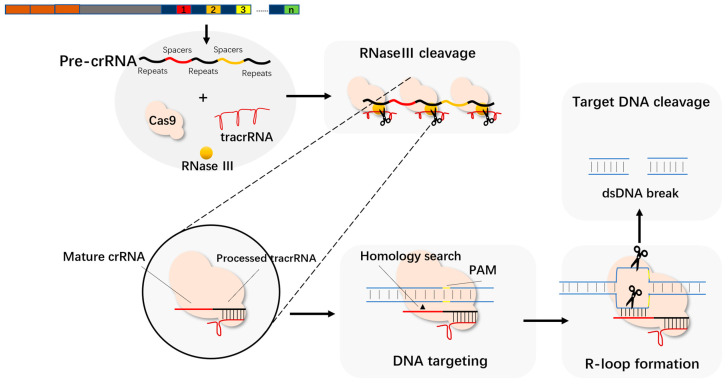
TracrRNA and mature crRNA form a dimer to guide the cleavage of the target sequence.

Cas9, as an endonuclease, contains two central HNH and RuvC that exert endonuclease activity. Jinek et al. found that Cas9 has a strong ability to cleave DNA under the mediation of tracrRNA and crRNA [21]. When the CRISPR/Cas system works, tracrRNA forms double-stranded RNA with pre-crRNA through complementary base pairing and assembles into a complex with the protein encoded by cas9. The spacer sequence is then cleaved under the action of RNase III, and finally a crRNA containing a spacer sequence RNA and a partial repeat sequence is formed. This complex binds to the target DNA under the guidance of crRNA, and then the two endonuclease active sites of Cas9 cut the DNA double-strand, in which the HNH site cuts one strand complementary to the crRNA, and RuvC cuts the other strand (Figure 3).

Moreover, crRNA and part of the tracrRNA also play the role of guiding Cas9 when they are fused into single guide RNA (sgRNA) [21]. People use artificially designed sgRNA to guide Cas9 gene modification, and have performed various far-reaching applications, including knock-out [22,23], knock-in [24], gene repression or activation [24,25], multiplex editing [24] and functional genomic screens.

When it comes to the application of the CRISPR/Cas9 system in pathogen detection, we must mention the work of Pardee et al. [26]. As mentioned above, the Cas9 protein can recognize PAM (protospacer adjacent motif) under the guidance of gRNA, and then exercise its ability to cleave (this is called cis-cleavage). They used the specific cleavage ability of Cas9 on different target sequences, combined with an isothermal RNA amplification technique called nucleic acid sequence-based amplification (NASBA) and the color reaction of filter paper (toehold activation) and developed a low-cost RNA virus detection method (NASBA-CRISPR cleavage, NASBACC). In this method, RNA is reverse-transcribed by NASBA, amplified to obtain dsDNA, and then Cas9 specifically recognizes and cuts dsDNA, and the signal is amplified by the filter-paper color reaction, which means it can accurately detect the Zika virus and use Cas9 to determine single-nucleotide polymorphisms to distinguish different strains with a sensitivity of 1 fM.

Wang et al. developed a Cas9 nickase-based amplification reaction (Cas9nAR) method. Cas9nAR uses a sgRNA-Cas9n complex with single-stranded nicking properties, a strand-displacing DNA polymerase and two primers with Cas9n cleavage sequences, through the cyclic process of initiation, extension, nicking and replacement reactions. The target sequence in the sample genomic DNA is amplified at a constant temperature (37 °C). In a sensitivity test for the detection of Salmonella typhimurium, a detection limit similar to that of qPCR was achieved [27]. Although this method has the advantage of amplifying the target sequence without length limitation, compared to NASBACC, its operation is cumbersome, and it is more difficult to apply in practice.

In addition, the two active sites of Cas9 were completely inactivated by amino acid mutation, and the resulting deactivated Cas9 (dCas9) still had the ability to bind target DNA under the guidance of sgRNA. Zhang et al. split the luciferase into two halves and then combined it with the dCas9 protein and designed a pair of sgRNAs targeting the upstream and downstream regions of the target DNA. When the dCas9 on both sides recognizes the target DNA and is adjacent, the luciferase activity is activated and emits a highly enhanced fluorescence signal, allowing for the detection of Mycobacterium tuberculosis [28]. Based on this property, several DNA detection methods have been developed (Table 1).

### 3.2. Cas12a

**Figure 4:**
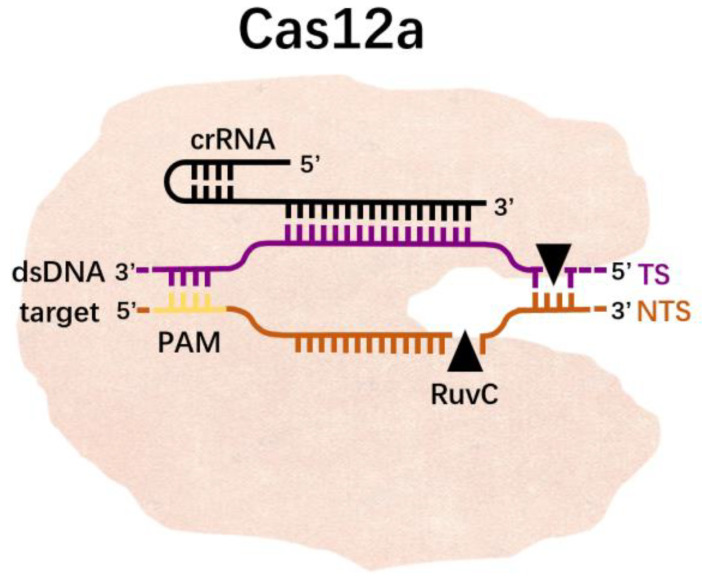
Cas12a-crRNA complex binds a dsDNA substrate and generates a 5′-overhang staggered cut by using a single RuvC nuclease.

CRISPR/Cas12a is a member of the Class II CRISPR/Cas system [36]. The CRISPR/Cas12a system contains the Cas12a protein and a shorter CRISPR RNA (crRNA). Unlike Cas9, Cas12a does not require the assistance of RNA or other proteins in the process of processing pre-crRNA into mature crRNA, nor does it require RNase III [37]. Therefore, Cas12a can achieve the cleavage of the target without tracrRNA. Cas12a identifies double-stranded DNA (dsDNA) via a single crRNA, thereby inducing staggered DNA breaks on the non-targeting and targeting strands via the RuvC and Nuc endonuclease domains, respectively [38]. Cas12a recognizes the double-stranded DNA of T (thymine)-rich PAM under the guidance of crRNA, and then unzips the double-stranded DNA. After melting, the target strand in the double-stranded DNA is complementary to the crRNA, causing a conformational change, exposing the RuvC site, which cuts the non-target strand (NTS) in the double-stranded DNA, and then cuts the TS; this is referred to as the cis-cleavage activity of Cas12a. After cleavage, the Cas12a-crRNA complex remains bound to the target dsDNA, the NTS is trimmed and the cleavage product is released after cleavage, which leaves the still active RuvC site exposed and able to bind to ssDNA and exert trans-cleavage activity (Figure 4). Therefore, the specificity of Cas12a can be guaranteed by designing crRNA complementary to the target sequence. Then, combined with fluorescent probes or immunochromatography technology, nucleic acid detection can be achieved.

**Figure 5:**
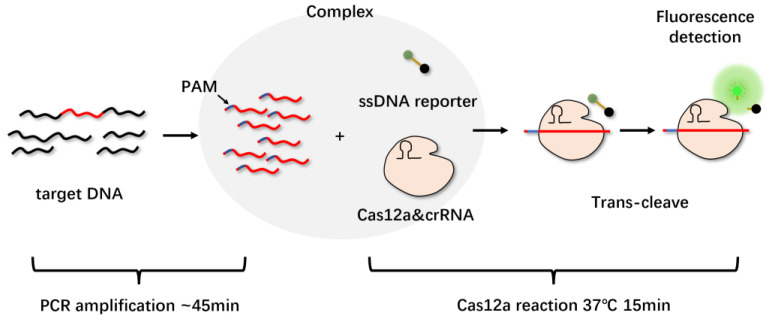
HOLMES detection platform. The target DNA was specifically amplified by PCR or other amplification methods, and crRNAs were designed to target somewhere in the target DNA. The PAM sequence was designed on the primer, which could be introduced during amplification. The CRISPR/Cas12a-crRNA was then mixed with the amplified product to form a ternary complex if the target DNA was present. The quenched reporter was then cleaved in trans, activating the fluorescence.

Although CRISPR/Cas12a can be directly activated using unamplified target DNA in a sample, amplifying pathogenic nucleic acids to further improve its sensitivity is necessary. Li et al. integrated quenched fluorescent single-stranded DNA reporter probes with PCR amplification to develop a HOLMES detection platform for the rapid detection of target DNA and RNA (Figure 5). Before PCR amplification was added, the minimum detection limit was 0.1 nM, and after combining with PCR, the detection limit could be as low as 10 aM [39].

In addition to the combination of PCR technology, the introduction of isothermal nucleic acid amplification technology eliminates the dependence on high-end temperature control equipment, allowing for the detection method to be used beyond the laboratory and making it possible to implement on-site. Combining Cas12a single-stranded DNase activation with isothermal amplification, Chen et al. developed a method called DNA endonuclease-targeted CRISPR trans reporter (DETECTR); this method can identify HPV16 and 18 types in patient samples within one hour, and the method achieves the attomolar sensitivity of DNA detection [40]. Li et al. applied recombinase-mediated isothermal nucleic acid amplification to this system and established a simple and high-sensitivity detection platform for Listeria monocytogenes, with the detection limit reaching 26 cfu/mL [41]. Zhang et al. also combined recombinase polymerase amplification (RPA) technology with CRISPR/Cas12a to detect SARS-CoV-2 [42].

To make the entire system more convenient and faster, the researchers also added immunochromatographic test strips to visualize the detection results. Wang et al. developed a fast, sensitive, instrument-free ASFV detection method (CRISPR/Cas12a-LFD) based on CRISPR/Cas12a technology and lateral flow detection, wherein ssDNA reporters labeled with quenching fluorescent molecules or digoxigenin and biotin were used for fluorescence and lateral flow detection, respectively. The method is able to complete the entire process from sampling to result reading in one hour, and the sensitivity can reach 20 copies/reaction [43].

Some scholars have also made attempts at signal conversion of the detection system; Liu et al. utilized the signal-amplifying ability of Cas12a and simultaneously used l-methionine stabilized gold nanoclusters as efficient ECL emitters to achieve ECL signal transduction. The method can complete the detection of HPV16 within 70 min, and its detection limit can reach 0.48 pM [44].

Due to its robust sensitivity and operability, Cas12a has also become increasingly active in pathogen detection applications (Table 2).

In addition to the above example of Cas12a as a signal sensor, scientists are also working on other easier and more efficient methods. Shen et al. also integrated magnetic nanoparticles with the system to detect Salmonella, which performed well in spiked chicken samples [55].

### 3.3. Cas12b

Like Cas12a, Cas12b, which is also from the Class II, Type V family, has trans-cleavage activity. That is, under the guidance of sgRNA, Cas12b can activate the ability to cleave any ssDNA in the system after it specifically recognizes and binds to the target DNA in the form of complementary base pairing. It was later found that trans-cleavage could be triggered regardless of whether the target is ssDNA or dsDNA. When targeting ssDNA, there is no cleavage site restriction (no PAM required), and the cleavage is faster; however, when dsDNA is used as the target, for Cas12b to perform efficient trans-cleavage, the target dsDNA is required to contain PAM. Based on the trans-cleavage characteristics of Cas12b, Li et al. developed a rapid nucleic acid detection method by combining Cas12b with LAMP isothermal amplification technology and named it HOLMESv2. This method can achieve a detection limit of 10^−8^ nM for the target, and the specificity reaches a single base [56]. Although they followed up with a one-step HOLMESv2 attempt, the sensitivity was much lower than that of the two-step method, which is also a possible direction for improvement in the future.

In recent years, there have also been studies on Cas12b, for example, Sam et al. combined LAMP and Cas12b detection to develop a DNA detection platform for Mycobacterium tuberculosis, named tb-QUICK, with a detection limit of 1.3 copies/μL in two hours [57]. Huang et al. constructed a Cas12b-based detection system for Campylobacter jejuni, and determined the detection limit of the system to be 11 copies/μL by simulating contamination [58]. However, this study took too much time in the early sample processing and nucleic acid extraction stage, which is also a problem that must be considered when attempting rapid nucleic acid detection.

### 3.4. Cas13a

Like other Class II system effector proteins, Cas13a also has a bilobal structure, but it also shows different structural features. It includes a REC flap with Helical-1 domain and a NUC flap with two HEPN domains, as well as two RNase catalytic pockets responsible for cleaving Pre-crRNA and target RNA which are located on Helical-1 and HEPN domains, respectively. When crRNA binds to it to induce significant conformational changes, two conserved HEPN domains form an external catalytic site for cleavage of the target RNA [59].

**Figure 6:**
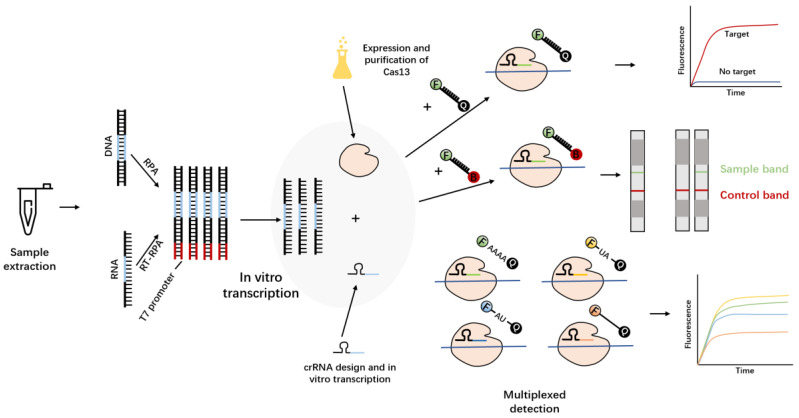
Complete SHERLOCK experimental workflow. The substrate DNA or RNA was first amplified by RPA or RT-RPA to increase the substrate concentration. Subsequently, T7 transcriptase transcribes the amplified DNA into RNA and mixes it with Cas13a–crRNA reaction solution. According to different reporters, lateral flow test strips or fluorescent signals can be selected as the output mode. Four Cas proteins with different cutting preferences can also be used for multiple detection.

As early as 2016, Abudayyeh, working in Zhang Feng’s team, proposed, for the first time, that Cas13a (C2c2) has a trans-cleavage activity that does not depend on the nucleic acid sequence [60]. The so-called trans-cleavage activity of Cas13a means that Cas13a forms a ternary complex with the target RNA under the guidance of crRNA, and then realizes the cleavage of any ssRNA in the system. Kellner and others, in the same team, took advantage of this cleavage activity to develop a nucleic acid detection method, named specific high-sensitivity enzymatic reporter unlocking (SHERLOCK) [61,62] (Figure 6). This is also the first time upon which the trans-cleavage activity of the Cas protein has been used to achieve in vitro nucleic acid detection, showing high sensitivity at the aM level and single-base resolution in the detection of the Zika virus and the dengue virus. Recently, some researchers have applied this technology more widely (Table 3).

Although the first-generation SHERLOCK platform has been widely studied and applied, it still has some limitations. For example, the first generation of SHERLOCK can qualitatively detect nucleic acids but cannot provide quantitative data, and relies more on fluorescence reading equipment. To improve the detection performance, Feng’s team made improvements to the SHERLOCK system and created the second-generation SHERLOCK system—SHERLOCKv2 [63]. They used four Cas proteins with different cleavage preferences (LwaCas13a, PsmCas13b, CcaCas13b and AsCas12a) and individually designed specific reporters for them to detect the four viruses simultaneously. The researchers also found that the Cas13 cleavage product activates another Cas protein, namely Csm6,which can amplify the detection signal and further enhance the sensitivity of this method. The second single-stranded RNA structure can be cleaved by activated Csm6, which enhances the signal relative to background, improving the kinetics of the SHERLOCKv2 reaction, even achieving results without RPA amplification; compared to the first-generation method, SHERLOCKv2 uses far fewer primers in the pre-amplification step. The modified SHERLOCKv2 is not limited to the output of fluorescent signals, but can also be applied to test strip detection, which makes SHERLOCKv2 very easy to use. Provided that there is a sample, SHERLOCKv2 can play a role in the field.

**Table 3:** More applications of CRISPR/Cas13a in pathogen detection.

Cas Protein	Pathogen	Platform Name	Amplification Methods	Visualization	Sensitivity	Time	References
LwCas13a	PPRRSV	SHERLOCK	RPA	Eye/LFD	172 copies/μL	<1 h	[64]
LwCas13a	BVDV	SHERLOCK	RT-RPA	Fluorescence	103 pM	-	[65]
LwCas13a	*Staphylococcus aureus*	CCB-Detection	PCR/T7transcription	Fluorescence	1 CFU/mL	<4 h	[66]
LwCas13a	H7N9	-	RT-RPA	Fluorescence	1 fM	50 min	[67]
LwCas13a	*Feline calicivirus* (FCV)	-	RPA	Fluorescence/LFD	5.5 copies/μL	-	[68]
LwCas13a	TMUV	-	RPA	Fluorescence	100 copies/μL	50 min	[69]
LwCas13a	ASFV	CRISPR/Cas13a–LFD	RAA	LFD	10 copies/μL	<2 h	[70]
LwCas13a	EMCV	-	RAA	LFD	10 copies/μL	<1 h	[71]
LwCas13a	*P. vivax*/*P. falciparum*	SHERLOCK	RPA	Fluorescence	10 aM	-	[72]
LwCas13a	HBV	-	RCA/PCR	Fluorescence	1 copies/μL	-	[73]

In nucleic acid detection, the sensing method of the signal is also particularly important. Due to the characteristics of low background noise and high signal output efficiency using fluorescence as a signal sensing method, various researchers have also used smartphones [74], microfluidic chips [75] and portable detectors [76] to record fluorescent signals. In addition to advances in fluorescence sensing, Heo et al. utilized reporter RNA (reRNA)-coupled electrochemical sensors as a signal output method. After the crRNA-Cas13a complex activates the activity of trans-cleavage through viral RNA, the cleavage of the reRNA immobilized on the electrode changes the current passing through the electrode, and then reading the current change can quantify the presence of the virus [77]. Liu et al. combined CRISPR/Cas13a with plasmon-enhanced fluorescence. Only reRNAs cleaved by Cas13a without activation could bind to plasmonic fluorescence; therefore, a higher signal intensity indicates that the amount of target RNA present in the original sample is lower. This signal-boosting method shows an almost 100-fold lower limit of detection.

### 3.5. Cas14a

**Figure 7:**
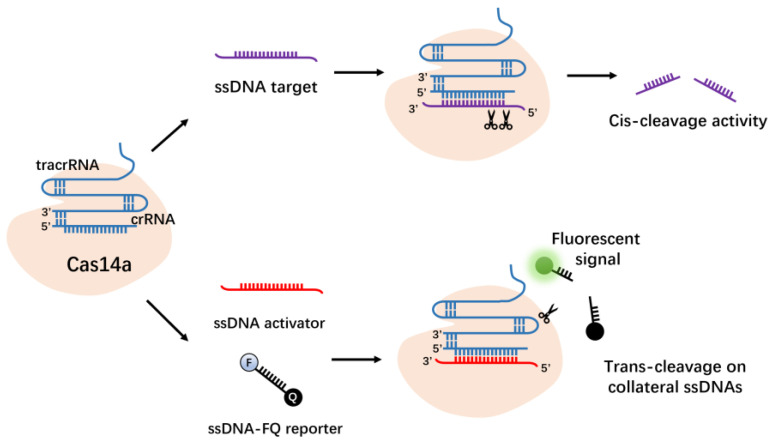
CRISPR–Cas14a has cis- and trans-cleavage activity on single-stranded DNA.

Cas14 is a new DNA-targeting CRISPRs effector protein identified by Doudna et al. in the archaea “DPANN” phylum [78]. Cas14a, like Cas12a, is from the Type V family of the Class 2 system. Cas14a is the smallest Class 2 CRISPRs effector demonstrated to date, containing approximately 400–700 amino acids, half of the Cas9 protein (950–1400 amino acids). Akin to Cas12a, Cas14a also possesses RNA-guided ssDNA-targeting endonuclease activity (Figure 7). The Cas14a protein does not need to recognize the PAM site in the DNA sequence. By combining the non-specific single-stranded DNase activity of the Cas14 protein with isothermal amplification technology, it may be used for high-fidelity DNA single-nucleotide polymorphism genotyping and ssDNA virus detection [79].

### 3.6. Application of the CRISPR System in Pathogen Detection

We list the CRISPR/Cas proteins in the text and tabulate some of their properties for easy reading (Table 4).

## 4. Challenge

Nucleic acid detection methods based on CRISPR/Cas biosensors have the advantages of strong specificity, high sensitivity and simple operation, and do not require instruments under some conditions. These methods can detect even trace amounts of virus and distinguish between different subtypes or mutations. They can also be integrated with various technologies to suit the needs of different scenarios. However, these techniques still have some problems (described next) that cannot be ignored.

### 4.1. Sequence Restriction

Some CRISPR/Cas effector proteins, such as CRISPR/Cas12a, require PAM sequences to identify target dsDNA. On the one hand, this feature enhances the specificity of target recognition, but on the other hand, it also limits the range of selectable target sequences. Therefore, when detecting shorter sequences, or identifying single-nucleotide polymorphisms, there may be less room for selection, limiting its application. To reduce the dependence on the PAM sequence, the PAM sequence was introduced into the PCR product using primers containing PAM in HOLMES, so that HOLMES can detect dsDNA independently of the PAM sequence [39]. Wang et al. also used the LAMP amplification method to design the core primer containing the PAM site, allowing for the LAMP amplicon to contain a specific PAM site for CRISPR/Cas12a recognition. This method can thus detect any target sequence, even without targets containing PAM sites, as long as the design requirements of LAMP are met [80].

### 4.2. Multiplexing and Quantitative Detection

SHERLOCKv2 uses four Cas proteins with different cleavage preferences to cleave individually designed reporters, enabling multiplex detection [62]; however, this has strict requirements on the amount of Cas protein in the system, and different Cas proteins and reporters may cross-cut, thus affecting the results. Therefore, it will be more conducive to multiple detection to find Cas effector proteins with different trans-cutting preferences and avoid Cas effector proteins cutting the same reporter or Cas effector proteins cutting different reporters at the same time. Ackerman et al. also proposed a multiplex virus detection method combining a microwell array with CRISPR/Cas, called ARMEN (arrayed reactions for multiplexed evaluation of nucleic acids); this method can simultaneously distinguish at least 10 related viruses among 169 human diseases with published genome sequences and can identify subtypes of influenza A strains [81]. Welch et al. also introduced microfluidic technology and developed microfluidic combinatorial arrayed reactions for the multiplexed evaluation of nucleic acids, which has been used to detect SARS-CoV-2 [82].

In terms of quantitative detection, the amplification product can easily reach a saturated state due to the high amplification efficiency of the introduced amplification method, and the limitation of reporters makes it difficult to quantify high-concentration targets. Before the introduction of CRISPR/Cas effectors, it is generally necessary to pre-amplify the sample nucleic acid. This step also affects the true concentration of the original sample, making quantitative detection difficult.

### 4.3. Sample Pre-Treatment

How to pre-treat samples rapidly and without contamination is the key to the nucleic acid detection process, especially for complex samples. Most CRISPR/Cas-based pathogen detection methods have pre-processing steps for the original sample. In vitro amplification of nucleic acid may introduce a base mutation of the target sequence due to the fidelity of DNA polymerase, thus interfering with the detection results. Shinoda et al. employed CRISPR/Cas13 and microarray technology to detect non-amplified RNA directly [83]. Although the quantification of nucleic acids is achieved due to the absence of amplification, the sensitivity is reduced compared to pre-amplification followed by detection (fM). In addition, the results of targeted amplification may be false positive due to aerosol pollution. Therefore, in the future, we should look for easier nucleic acid extraction methods to save time, and try to directly detect non-amplifying targets for quantification, find a balance between the two or determine a strong Cas effector protein that is more suitable for complex sample environments without amplification.

### 4.4. Contamination during Operation

When the CRISPR/Cas system is used for nucleic acid detection, a two-step method is generally used. The first step is to conduct nucleic acid pre-amplification, and the second step is to add effector protein complexes and reporters. This may expose the reaction system to RNase in the air during the second step, resulting in contamination and affecting the result. To avoid this, Li et al. integrated the target pre-amplification and biosensing stage in a one-step reaction to achieve the detection of SARS-CoV-2 [84]. Therefore, future research will also explore this aspect, which can not only simplify the operation, but also achieve the purpose of reducing pollution.

### 4.5. On-Site Deployment

Although most of the pathogen detection technologies based on the CRISPR/Cas system are in the research and development stage, they will eventually be applied in practice. How to design this technique to make it more suitable for practical deployment is also a suggested direction for future research. In addition to the efforts in sample handling, avoidance of contamination and quantitative detection, more efficient signal-reading techniques also need to be discovered. Finally, efforts should be made to develop detection methods with strong specificity, high sensitivity, simple operation, controllable costs and simple reading, which are more suitable for practical deployment.

### 4.6. The Lack of a Uniform Standard

The use of CRISPR/Cas biosensing technology for pathogen detection remains in the research and development stage, and many aspects remain in the exploratory stage, such as the concentration of each component in the reaction system, reaction time, reaction temperature, nucleic acid extraction method and signal output method (each of which will affect the result). For this technique to be used in point-of-care testing, food safety monitoring, etc., it is necessary to customize standards or specifications that conform to practical applications.

## 5. Summary

Pathogenic microorganisms present in nature seriously threaten public health and the global economy. Therefore, the development of rapid, sensitive, specific, economical and field-applicable pathogen diagnosis methods can help control and prevent disease transmission. The CRISPR/Cas system is not only an excellent gene-editing tool, but also a powerful diagnostic technology. For example, techniques for targeting DNA using the recognition and cleavage capabilities of CRISPR/Cas9 effectors have been developed for molecular diagnostics. Unlike CRISPR/Cas9, CRISPR/Cas12 and CRISPR/Cas13 have been applied in biosensing scenarios due to their trans-cutting ability, creating a new era of molecular diagnosis. Although the pathogen detection technique based on the CRISPR/Cas system has the advantages of rapid detection, low cost and wide applicability, there are still many areas that need to be improved before the actual performance in the complex environments encountered in practice can be accepted, for example: find more efficient methods for original sample processing and simplify nucleic acid extraction steps; to realize multiple detection and quantification of several pathogens; to avoid contamination during the Cas protein effect stage. In conclusion, a pathogen detection technology based on the CRISPR/Cas system provides a new means for pathogen detection molecular methods, and it is now effective and developing rapidly. It is foreseeable that when various disciplines such as materials, communication and AI are applied thereto, the pathogen detection technology based on the CRISPR/Cas system will become a more promising and widely used in vitro pathogen-detection tool.

## Figures and Tables

**Table 1:** More applications of CRISPR/Cas9 in pathogen detection.

Cas Protein	Pathogen Type	Pathogen	Visualization	Sensitivity	Time	References
Cas9	Viruses	*Zika*	Colorimetry	1 fM	2–3h	[26]
Cas9	Bacteria	*Listeria monocytogenes*	LFA	150–200 copies/μL	2 h	[29]
Cas9	Bacteria	*E. coli*	SDA/RCA	40 CFU/mL	2–3 h	[30]
Cas9n	Bacteria	*S. typhimurium*	Fluorescence	2 copies/μL	<1 h	[27]
dCas9	Bacteria	*Mycobacterium* *tuberculosis*	Bioluminescence	5 × 10^−5^ nmol/mL	<1 h	[31]
dCas9	Bacteria	Methicillin-resistant *Staphylococcus aureus* (MRSA)	Fluorescence	10 CFU/mL	<0.5 h	[32]
dCas9	Bacteria	*Scrub typhus* (ST)/severe fever with thrombocytopenia syndrome (SFTS)	SMR biosensor	0.54 aM/0.63 aM	0.5 h	[33]
dCas9	Viruses	HPV	Microplate reader/eye	-	<0.5 h	[34]
dCas9	Bacteria	*Acinetobacter baumannii*/*Klebsiella pneumoniae*	Spectrometry	10^−5^ mol/L	1 h	[35]

**Table 2:** More applications of CRISPR/Cas12a in pathogen detection.

Cas Protein	Pathogen	Platform Name	Amplification Methods	Visualization	Sensitivity	Time	References
LbCas12a	ASFV	POC	RPA/LAMP	Fluorescence	100 fmol	<2 h	[45]
Cas12a	ASFV	LAMP-CRISPR	LAMP	Fluorescence	7 copies/μL	<1 h	[46]
Cas12a	Yersinia pestis	Cas12a-UPTLFA	RPA	UPT-LFA	3 aM	<1 h	[47]
Cas12a	PRRSV	-	RT-RPA	ssDNA-FQ	1 copies/μL	25 min	[48]
Cas12a	Listeria monocytogenes	Cas12aFDet	PCR/RAA	Fluorescence	0.64 aM	15 min	[49]
Cas12a	Vibrio parahaemolyticus	-	RPA	Eye	10^−18^ M	<30 min	[50]
Cas12a	Toxoplasma gondii	RAA-Cas12a-Tg	RAA	ssDNA-FQ	1 fM	1 h	[51]
Cas12a	Staphylococcus aureus	RAA-Cas12a	RAA	Fluorescence	10 copies/μL	1 h	[52]
Cas12a	pathogenic Yersinia enterocolitica	-	RPA	Eye	1.7 CFU/mL	45 min	[53]
Cas12a	Leptospira	-	RPA	Fluorescence/LFDA	100 cells/mL	<2 h	[54]

**Table 4:** Application of the CRISPR system in pathogen detection.

Cas Protein		Detection Platform	Guide RNA	Target Type	Trans-Cleavage Activity	Amplification Methods	Sensitivity
**Cas9**			sgRNA	DNA	No	CAS-EXPAR	aM (10^−18^)
Cas12	Cas12a	HOLMES	crRNA	DNA	Yes	PCR/RT-PCR	aM
	Cas12a	DETECTR	crRNA	DNA	Yes	RPA	aM
	Cas12b	HOLMESv2	sgRNA	DNA	Yes	LAMP	aM
Cas13	Cas13a	SHERLOCK	crRNA	RNA	Yes	RPA	aM
	Cas13b	SHERLOCKv2	crRNA	RNA	Yes	RPA	zM (10^−21^)
Cas14	Cas14a	DETECTR	sgRNA	ssDNA	Yes	RPA	aM

## Data Availability

Not applicable.
